# The effect of systemic hypertension on prostatic artery resistive indices in patients with benign prostate enlargement

**DOI:** 10.1016/j.prnil.2022.09.001

**Published:** 2022-09-27

**Authors:** Stephen O. Onigbinde, Christianah M. Asaleye, Abdulkadir A. Salako, Bukunmi M. Idowu, Abimbola O. Onigbinde, Adeyinka Laoye

**Affiliations:** a Department of Anatomical Sciences, School of Medicine, St George's University, Grenada; b Department of Radiology, Obafemi Awolowo University and Obafemi Awolowo University Teaching Hospitals Complex, Ile-Ife, Osun State, Nigeria; c Department of Surgery, Obafemi Awolowo University and Obafemi Awolowo University Teaching Hospitals Complex, Ile-Ife, Osun State, Nigeria; d Department of Radiology, Union Diagnostics and Clinical Services Plc, Yaba, Lagos, Nigeria; e Department of Physiology, Neurology and Behavioral Sciences, School of Medicine, St George's University, Grenada; f Urology Department, Doncaster and Bassetlaw Teaching Hospitals NHS Found, South Yorkshire, United Kingdom

**Keywords:** Benign prostatic enlargement, Doppler ultrasonography, Hypertension, Resistive index

## Abstract

**Background:**

To investigate the effect of systemic hypertension on the prostatic artery resistive indices by a comparative ultrasonographic evaluation of the prostate gland in normotensive and hypertensive patients with benign prostatic enlargement (BPE).

**Materials and methods:**

The participants had BPE and presented at the outpatient urologic clinic of a tertiary hospital. They were divided into normotensive and hypertensive groups. Each group had fifty patients. Calculation of international prostate symptom score, measurement of blood pressure, and transrectal ultrasonographic evaluation were done.

**Results:**

The mean age for the normotensive and hypertensive groups were 66.9 ± 9.8 and 66.0 ± 10.7 years, respectively (*P* = 0.662). Patients with hypertensive BPE had a significantly higher mean transitional zone volume, transitional zone index, presumed circle area ratio, quality of life score, and prostatic arterial resistive indices than the age-matched normotensive BPE patients.

**Conclusion:**

Patients with BPE and with hypertension had significantly higher prostate arteries resistive indices than normotensives with BPE. Even in patients with BPE and controlled hypertension, the prostatic artery resistance indices were still elevated than that of normotensive men with BPE.

## 1 Introduction

Histologically, benign prostatic hyperplasia is defined by the presence of non-cancerous nodules composed of smooth muscle and epithelial cells inside the prostatic transitional zone (TZ).^1^ Benign prostatic enlargement (BPE) is the non-malignant increase in the size of the prostate gland and is the second most common indication for surgery in men >60 years old.^2^^,^^3^ It is the most common benign pathological condition affecting elderly men.

The global burden of BPE outweighs that of all other male genitourinary diseases combined.^4^^,^^5^ In 2019, an estimated 11.26 million men were newly diagnosed with BPE throughout the world.^6^ The lifetime prevalence of BPE is 22.8–29.6%.^7^^,^^8^

Clinical BPE is diagnosed when at least two of these criteria are present: moderate to severe lower urinary tract symptoms (LUTS) with an international prostate symptom score (IPSS) >8, prostatomegaly with prostatic volume >30 mL, and diminished maximum urinary flow rate (Qmax) less than 15 mL/s.^2^

Systemic hypertension is a global health problem, accounting for substantial morbidity and mortality from heart disease, stroke, and renal failure.^9^^,^^10^ Globally, as of 2019, there were more than 1 billion people with systemic hypertension.^11^ Hypertension is one of the common comorbidities of BPE in the elderly.^12^ Over 25% of elderly men with BPE also have hypertension.^13^

Transrectal ultrasound (TRUS) is the standard first-line investigation for prostatic pathologies because of the increased proximity of the transducer to the prostate gland, reduced artifactual interference, improved resolution, and generation of detailed zonal anatomical information.^14^, ^15^, ^16^, ^17^, ^18^

In recent years, the prostatic resistive index (PRI) measured by power Doppler imaging has been used to evaluate patients with BPE.^19^ It has been shown that the PRI is increased in patients with BPE and it is related to the severity of bladder outlet obstruction.^19^, ^20^, ^21^

A previous study revealed that men with hypertension are predisposed to more severe LUTS and larger prostatomegaly than men without high blood pressure (BP).^22^ In addition, Berger et al indicated that diabetes mellitus might be a risk factor for BPE.^23^ Chen et al reported a very weak but significant positive correlation (r = 0.23, *P* < 0.05) between the resistive indices of the periurethral arteries and right neurovascular bundles of the prostate and some cardiovascular risk factors (hypertension, diabetes, hyperlipidemia, obesity, and a history of cardiovascular events).^24^ Similar findings were also documented by Baykam et al.^25^

The aim of this study was to find out if there is any significant difference between the prostatic arteries resistive indices of subjects with BPE co-existing with hypertension and that of normotensive subjects with BPE. We hypothesized that the prostate arteries resistive indices would be significantly higher BPE patients with hypertension than normotensives with BPE.

## 2 Materials and methods

This was a descriptive comparative study. The participants were hypertensive and normotensive men, aged 40–90 years, with LUTS and a clinical diagnosis of BPE. They were recruited consecutively from the Urology clinic of our tertiary hospital between October 2017 and September 2018. The hospital's Ethics and Research Committee approved the study protocol (ERC/2017/01/01). Written informed consent was obtained from all the participants.

The study included individuals who were diagnosed with BPE based on clinical history, digital rectal examination, PSA testing (<10 ng/ml), and transabdominal prostatic ultrasound (with prostate gland volume >25 cm^3^ on TRUS). They were categorized into two–hypertensive and normotensives–each group comprised fifty men.

The participants' BP was taken while seated, after he had rested for at least 15 minutes prior to the measurement. Three readings were taken and averaged. Systemic hypertension was defined as a BP ≥140/90 mmHg or an elevated BP necessitating antihypertensive therapy.^26^

The body mass index (BMI) was computed by dividing the weight (Kg) by the square of the height (meters). Participants with BMI of 18.5–24.9 kg/m^2^, 25.0–29.9 kg/m^2^, 30–39.9 kg/m^2^, and >40 kg/m^2^ were classified as normal, overweight, obese, and morbidly obese.^27^

Participants with hypertension placed on beta blockers, alpha-1 adrenoceptors blockers (prazosin, doxazosin, tamsulosin, etc), and phosphodiesterase-5 inhibitors (tadalafil) were excluded because they can affect the severity of LUTS. Other exclusion criteria include severe hydronephrosis or serum creatinine >132.5 μmol/L, PSA level of >10 ng/ml, diabetes mellitus, hyperlipidemia, obesity (BMI ≥30 kg/m^2^), smoking, prostate or bladder cancer, prostatitis, bladder stone, urethral stricture, neurogenic bladder, acute urinary retention, history of transurethral resection of the prostate or previous urinary tract surgery, anal stenosis, and previous major rectal surgery.^19^

The sonographic measurements were carried out with a Mindray® model DC-7 ultrasound scanner with Doppler functionality (Shenzhen Mindray Bio-medical Electronics, Nanshan, Shenzhen, China). A transrectal biplanar transducer (frequency = 5.0–10.0 MHz) was used for TRUS scan of the prostate, while a curvilinear transducer (frequency = 3.5–5.0 MHz) was used for the transabdominal ultrasound scan of the prostate.

The total volume of the prostate gland was measured using transabdominal and TRUS techniques. All transabdominal measurements of the prostate were performed with full bladder. Measurements were performed with the participants in supine position. The longitudinal (LD), anteroposterior (APD), and transverse (TD) diameters of the prostate were measured. The volume of the prostate on transabdominal USS (TAPV) was calculated using ellipsoid formula (0.523 × LD × APD × TD).^19^

Transrectal sonography was done with the patients lying in the left lateral decubitus position. Multiple transverse and sagittal sections were obtained and recorded after visualizing the TZ and whole prostate outline. The TD and the APD of the whole prostate and the transition zone, at the largest cross sectional area, were obtained. Also, the craniocaudal diameters (CCD) of the whole prostate and the TZ were measured on the midline sagittal images. Transrectal total prostatic volume and transitional zone volume (TZV) were also estimated using the ellipsoid formula (CCD × APD × TD × 0.523).^19^ The sonographic measurements were performed by the same sonologist three times and mean values derived in order to reduce intra-observer variability.

The IPSS, quality of life (QOL) score, maximum urine flow rate (Qmax), bladder wall thickness (BWT), transition zone index (TZI), presumed circle area ratio (PCAR), and postvoidal residual (PVR) urine volume, and triplex Doppler resistance indices (RI) of the right capsular artery (RIRCA), left capsular artery (RILCA), and urethral artery (RIUA) were calculated using previously published methods and guidelines.^19^^,^^28^

During power Doppler imaging, care was taken to minimize probe pressure on the rectal wall and an empty or nearly empty bladder was ensured so that compression effect by either the probe or full urinary bladder would not increase the intraprostatic pressure, which could alter the prostatic RI. The power Doppler gain was set to just below the noise threshold, so that blood flow was identified with minimum background noise and the low flow setting was used for optimal visualization of low flow intraprostatic vessels. Then, the pulsed waved spectral Doppler images were obtained from the urethral artery, right capsular artery, and left capsular artery on transverse section of the prostate. Care was taken to select Doppler measurements with angles of insonation of <60°. After the spectral Doppler wave form became stable, it was traced for three pulses. The peak systolic velocity, end-diastolic flow velocity, and RI were automatically calculated by the software of the ultrasound scanner. Three values for RI were measured for each of the three prostatic arteries and averaged to obtain the mean RI.

The data were analyzed using the IBM SPSS Statistics for Windows, version 22 (IBM Corp., Armonk, N.Y., USA). Means and standard deviation were gotten for the continuous variables (TPV, TZV, TZI, PCAR, RIRCA, RIUA, and RILCA). Categorical variables (age group, IPSS category, and QOL category) were presented as frequencies. The continuous data were non-parametric as determined by Kolmogorov–Smirnov test and Shapiro–Wilk test. Thus, Mann U Whitney test was used to compare the mean ranks of TPV, TZV, TZI, PCAR, BWT, PVR, IPSS, systolic blood pressure (SBP), diastolic blood pressure (DBP), QOL, RIRCA, RIUA, and RILCA between the two study groups. Correlation between RIRCA, RILCA, RIUA, SBP, and DBP was determined using Spearman's correlation. The correlation coefficients were classified as follows: r = 0–0.2 (very low, negligible, and probably meaningless correlation), r = >0.2–0.4 (low correlation which might warrant further investigation), r = >0.4–0.6 (moderate correlation), r = >0.6–0.8 (high correlation), and r = >0.8–1.0 (excellent/very high correlation).^29^ At 95% confidence interval, *P* ≤ 0.05 was considered statistically significant.

## 3 Results

The bio-demographic characteristics of the study population are shown in Table 1. There were one hundred participants in this study. Fifty were normotensive patients with BPE, while 50 were hypertensive patients with BPE. The hypertensive group did not significantly differ in age, height, weight and BMI from the normotensive group. The mean age for the normotensive and hypertensive groups were 66.9 ± 9.8 years and 66.0 ± 10.7 years, respectively (*P* = 0.66). There was no significant difference in the mean BMI of both groups. The BMI of the normotensive group was 23.7 ± 4.7 kg/m^2^, while that of the hypertensive group was 23.8 ± 4.4 kg/m^2^ (*P* = 0.98). The mean SBP of the hypertensive group was (129.52 ± 10.76) mmHg, while that of normotensive BPE participants was (125.4 ± 6.94) mmHg. The mean SBP was higher in the hypertensive group but the difference was not statistically significant (*P* = 0.087).

**Table 1:** Bio-demographic characteristics of study population

Variables		Normotensive BPE (*n* = 50)	Hypertensive BPE (*n* = 50)	Statistic	df	*P* value
**Age (years)**						
Range		46.0–88.0	43.0–89.0			
Mean ± SD		66.9 ± 9.8	66.0 ± 10.7	0.438	98	0.662a)
**Age Group (years)**						
*40–49*	Range	46.0–47.0	43.0–49.0	0.000	4	>0.999b)
	Frequency, *n* (%)	4 (8)	4 (8)			
*50–59*	Range	50.0–57.0	50.0–56.0			
	Frequency, *n* (%)	6 (12)	6 (12)			
*60–69*	Range	60.0–69.0	60.0–68.0			
	Frequency, *n* (%)	21 (42)	21 (42)			
*70–79*	Range	70.0–77.0	70.0–79.0			
	Frequency, *n* (%)	14 (28)	14 (28)			
*≥80*	Range	82.0–88.0	81.0–89.0			
	Frequency, *n* (%)	5 (10)	5 (10)			
**Height in (m)**						
Range		1.5–1.8	1.6–1.9			
Mean ± SD		1.7 ± 0.1	1.7 ± 0.1	−0.175	98	0.862a)
**Weight (kg)**						
Range		42.1–121.2	49.2–121.1			
Mean ± SD		70.7 ± 16.2	70.5 ± 14.9	0.090	98	0.928a)
**BMI (kg/m**^**2**^**)**						
Range		15.7–39.5	17.5–39.6			
Mean ± SD		23.7 ± 4.7	23.8 ± 4.4	−0.023	98	0.982a)

BMI, body mass index; BPE, benign prostatic enlargement.

a) Independent samples t-test was used to compare means.

b) Likelihood ratio test was used to compare proportions.

The mean RIRCA of the hypertensive BPE group was 0.7 ± 0.1, while that of the normotensive BPE group was also 0.7 ± 0.1. However, the mean rank of the hypertensive BPE group (56.6) was significantly higher than (*P* = 0.037) that of the normotensive BPE group (44.5) (Table 2).

**Table 2:** Resistive indices of the prostatic arteries

	Normotensive BPE group (*n* = 50)		Hypertensive BPE group (*n* = 50)		*U*	*P* value
	Mean ± S.D.	Mean Rank	Mean ± S.D.	Mean Rank		
RIRCA	0.7 ± 0.1	44.5	0.7 ± 0.1	56.5	947.500	**0.037**
RIUA	0.7 ± 0.1	38.7	0.7 ± 0.1	62.3	659.500	**<0.0001**
RILCA	0.6 ± 0.1	40.9	0.7 ± 0.1	60.1	769.500	**0.001**

BPE, benign prostatic enlargement; RIRCA, resistive index of the right capsular artery; RILCA, resistive index of the left capsular artery; RIUA, resistive index of the urethral artery.

The mean RIUA of the hypertensive BPE group was 0.7 ± 0.1, while the mean RIUA of the normotensive BPE group was also 0.7 ± 0.1. However, the mean rank of RIUA in the hypertensive BPE group (62.3) was significantly higher (*P* < 0.0001) than that of the normotensive BPE group (38.69) (Table 2).

The mean RILCA of the hypertensive BPE group subjects was 0.7 ± 0.1, while that of normotensive BPE subjects was 0.6 ± 0.1. The hypertensive BPE group (60.1) had a significantly higher (*P* = 0.001) mean RILCA than the normotensive BPE group (40.9) (Table 2).

The RIRCA and SBP had a low positive correlation in the normotensive BPE group (r = 0.34, *P* = 0.017) and a stronger moderate positive correlation in the hypertensive BPE group (r = 0.60, *P* < 0.0001). There was a low positive correlation between the RIRCA and DBP in the normotensive BPE group (r = 0.38, *P* = 0.006) and a stronger moderate positive correlation in the hypertensive BPE group (r = 0.49, *P* value < 0.001).

In the normotensive BPE group, RIUA yielded a low positive correlation with SBP (r = 0.34, *P* = 0.016), but yielded a stronger moderate positive correlation between RIUA and SBP (r = 0.60, *P* value < 0.001) in the hypertensive BPE group. Also, there was a low positive correlation between RIUA and DBP in the normotensive BPE group (r = 0.29, *P* = 0.039), but a stronger moderate positive correlation in the hypertensive BPE group (r = 0.49, *P* < 0.001).

The RILCA and SBP had a low positive correlation in the normotensive BPE group (r = 0.38, *P* = 0.007) and a stronger moderate positive correlation in the hypertensive BPE group (r = 0.48, *P* < 0.001). Correlation between RILCA and DBP was low positive in the normotensive BPE group (r = 0.37, *P* = 0.009), but moderately positive in the hypertensive BPE group (r = 0.55, *P* < 0.001).

Table 3 shows that the mean rank of the BWT, PCAR, TZI, and transition zone volume (TZV) of the hypertensive BPE group were significantly higher than that of the normotensive BPE group. There was no statistically significant difference between the TPV and PVR urine volume of the two groups.

**Table 3:** Comparison of B-mode ultrasound parameters

	Normotensive BPE group (*n* = 50)		Hypertensive BPE group (*n* = 50)		*U*	*P* value
	Mean ± S.D.	Mean Rank	Mean ± S.D.	Mean Rank		
TPV	48.5 ± 18.1	45.5	57.8 ± 26.3	55.5	1000.500	0.085
TZV	19.0 ± 12.2	44.0	27.5 ± 19.1	57.0	923.000	**0.024**
TZI	0.4 ± 0.1	44.0	0.4 ± 0.1	57.0	927.000	**0.026**
PCAR	0.7 ± 0.1	40.2	0.8 ± 0.1	61.0	737.000	**<0.0001**
BWT	2.8 ± 1.5	42.2	3.2 ± 1.3	58.8	835.000	**0.004**
PVR	56.1 ± 24.4	45.0	61.1 ± 24.2	56.1	970.000	0.054

BPE, benign prostatic enlargement; BWT, bladder wall thickness; PCAR, presumed circle area ratio; PVR, postvoidal residual urine; TPV, total prostate volume; TZI, transition zone index; TZV, transition zone volume.

The SBP, DBP, and IPSS yielded no statistically significant difference between the two study groups (Table 4). In contrast, the hypertensive with BPE group had a significantly higher QOL score than the normotensive with BPE group (Table 4).

**Table 4:** Comparison of clinical parameters

	Normotensive BPE group (*n* = 50)		Hypertensive BPE group (*n* = 50)		*U*	*P* value
	Mean ± S.D.	Mean Rank	Mean ± S.D.	Mean Rank		
SBP (mmHg)	125.4 ± 6.9	45.6	129.5 ± 10.8	55.5	1002	0.087
DBP (mmHg)	84.1 ± 4.6	46.0	87.1 ± 8.9	55.0	1.024	0.117
IPSS	16.0 ± 8.6	45.3	19.4 ± 8.1	55.7	990.5	0.073
QOL	3.4 ± 1.4	44.7	4.0 ± 1.1	56.3	961.5	**0.039**

BPE, benign prostatic enlargement; DBP, diastolic blood pressure; IPSS, international prostatic symptoms score; QOL, quality of life score; SBP, systolic blood pressure.

**Fig. 1:**
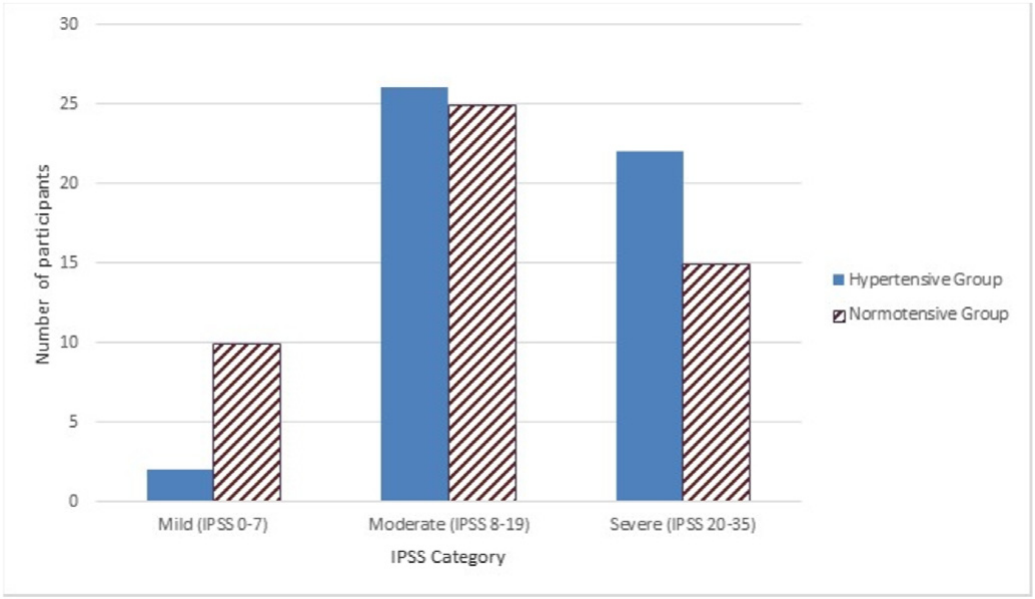
Clustered bar chart showing the distribution of subjects according to severity of lower urinary tract symptoms (LUTS) measured by the International Prostatic Symptoms Score (IPSS).

**Fig. 2:**
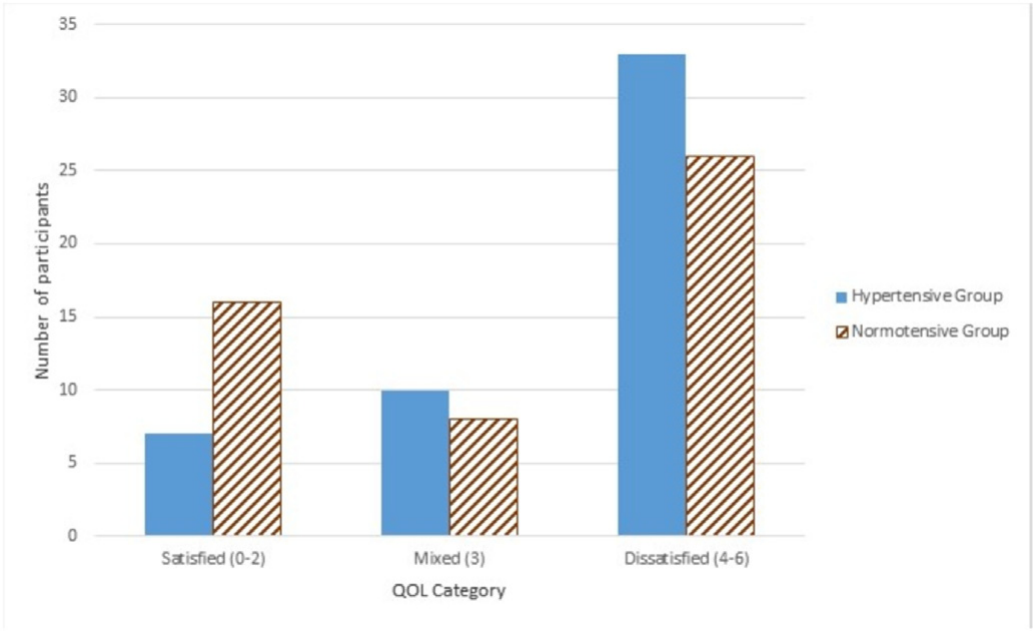
Clustered bar chart showing the distribution of subjects according to the quality of life score.

Fig. 1, Fig. 2 show the IPSS and QOL scores of the study participants.

## 4 Discussion

Increased arterial resistive indices in enlarged prostate glands have been thought to be due vascular compression against a rigid prostatic capsule, neuronally-mediated vasoconstriction, or vascular damage.^20^^,^^30^ Hypertension is associated with increased vascular damage and vasoconstriction.^31^

The RI can be used to quantify alterations in the blood flow of various target organs. In recent years, the PRI measured by Power Doppler Imaging has been used to evaluate patients with BPE.^13^^,^^19^ Several reports have shown that the PRI is increased in patients with BPE and it is related to the severity of bladder outlet obstruction.^13^^,^^19^ PRI was first proposed as a diagnostic tool to differentiate between BPE and normal patients by Kojima et al.^32^

In this study, the mean RI of the prostatic arteries was significantly higher in participants with hypertension than normotensives. For comparison, the mean RI of the urethral and capsular arteries was less than the values gotten by Abdelwahab et al^20^ in Egypt (RIRCA = 0.8 ± 0.1; RILCA = 0.8 ± 0.1; RIUA = 0.8 ± 0.1). The Nigerian patients in this study were chosen consecutively, while Abdelwahab et al did a random selection of 86 Egyptian patients. Their mean TPV (75.1 ± 44.7 ml) is larger than that of both groups (Normotensive = 48.5 ± 18.1 ml; Hypertensive = 57.8 ± 26.3 ml) in this study. The Egyptian patients had partially filled urinary bladders during TRUS–compression by the partially filled urinary bladders could have affected the resistive indices obtained.^13^ In addition, Abdelwahab et al did not exclude patients with systemic comorbidities such as DM which is known to affect the prostate gland.^23^

The increased mean RI values in the hypertensive group supports the finding by Michel et al^33^, in a study conducted among Germans, that there is an association between BPE and hypertension. This might be partly explained by a common pathophysiological factor such as an increased sympathetic nervous system activity in both BPE and hypertension. Although Baykam et al found no correlation between prostate arteries RI (PRI) and hypertension specifically; there was a correlation between PRI and metabolic syndrome.^25^ A review by Abdollah et al confirmed this association with metabolic syndrome.^34^

A similar pattern of increased PRI has also been reported in patients with diabetes mellitus. Berger et al reported that the RI of the TZ was significantly higher in BPE patients with type 2 diabetes mellitus, coronary artery disease, peripheral arterial occlusive disease than in healthy control patients.^23^^,^^35^ They surmised that vascular damage in the prostate induced prostatic enlargement which, in turn, accounts for the increased RI.

The mean IPSS (19.4 ± 8.1) and PVR (61.1 ± 24.2 ml) of hypertensives in this study were significantly higher than that of normotensives (IPSS = 16 ± 8.6; PVR = 56.1 ± 24.3 ml). This pattern is similar to findings by Sugaya et al^36^ and Michel et al^33^ The mean value of IPSS of the index study is close to what Michel et al^33^ got in their study of German patients, while our mean value of IPSS and PVR of hypertensives were significantly lower than the values reported by Sugaya et al.^36^ This disparity is probably because the hypertensives in this study and in the study by Michel et al enrolled those that had been commenced on antihypertensive medications. Sugaya et al^36^ studied Japanese BPE patients who were hypertensive and had not been commenced on antihypertensive medications. Güven et al^37^ in Turkey also found a positive correlation between benign prostate enlargement-related LUTS (storage and voiding symptoms) and SBP.

There was no statistically significant difference between the BPs of the two groups in this study. This lack of difference likely resulted from the fact that the BPE with hypertension enrolled had well-controlled BP (on antihypertensive medication). It would have been unethical to ask them to discontinue their medication just for the study. Therefore, we can conclude that even in patients with BPE and with controlled hypertension, the prostatic artery RIs are still elevated than that of normotensive men with BPE, as demonstrated by our results. The alternative would have been to recruit patients newly diagnosed with hypertension who had yet to commence antihypertensive treatment. Unfortunately, hypertension is highly prevalent in our environment with the age at first diagnosis often <40 years. At this age, significant BPE is unlikely to have developed.

In conclusion, the mean values of RIRCA, RIUA, and RILCA in hypertensive BPE patients were significantly higher than those of patients with normotensive BPE. The PRI showed low positive correlation with SBP and DBP in the normotensive group and moderate positive correlation with SBP and DBP in the hypertensive group.

A limitation of this study was that the influence of hypertension duration and antihypertensive-naivety (non-treatment or uncontrolled from drug resistance) on prostate blood flow could not be assessed. Also, this was a hospital-based study although we do not foresee a remarkable difference in PRI–hypertension relationship in a community-based study.

## Conflict of interest

All authors have no conflict of interest to declare.
